# Vertebroplasty for osteoporotic vertebral fractures: Additional concern on its usefulness

**DOI:** 10.4103/0019-5413.55980

**Published:** 2009

**Authors:** Viroj Wiwanitkit

**Affiliations:** *Wiwanitkit House, Bangkhae, Bangkok Thailand - 10 160*

Sir,

I read the recently published article by Diel *et al*. on the clinical value of vertebroplasty for osteoporotic vertebral fractures with great interest.^1^ I agree with the conclusion; however, there are some additional points to be concerned. The cost-effectiveness of verteboplasty on this specific purpose needs a systematic evaluation. Also, the turnaround time comparison should be performed. Finally, the availability of the technique must also be assessed. These will help clarify the exact usefulness of vertebroplasty.
